# Intracellular Behaviour of *Legionella* Non-*pneumophila* Strains within Three Amoeba Strains, Including *Willaertia magna* C2c Maky

**DOI:** 10.3390/pathogens10101350

**Published:** 2021-10-19

**Authors:** Aurélien Croze, Antoine Carlino, Benjamin Quélard, Adeline Saha, Tiphaine Convert, Jean-Baptiste Eberst, Sandrine Demanèche

**Affiliations:** R&D Department, Amoéba, 38 Avenue des Frères Montgolfier, 69680 Chassieu, France; a.croze@amoeba-nature.com (A.C.); a.carlino@amoeba-nature.com (A.C.); b.quelard@amoeba-nature.com (B.Q.); a.saha@amoeba-nature.com (A.S.); t.convert@amoeba-nature.com (T.C.); jb.eberst@amoeba-nature.com (J.-B.E.)

**Keywords:** free-living amoebae, *Legionella* non-*pneumophila*, intracellular pathogen, biological biocide

## Abstract

Legionellosis, an often-lethal pneumonia, is generally associated with contamination by *Legionella pneumophila*. This bacterium can persist in the environment and resist chemical treatment when it is internalized by amoebae. In addition, there is increasing medical proof that other *Legionella* species can be causative agents of Legionellosis. The objective of this study was to evaluate whether *Legionella* non-*pneumophila* (Lnp) strains were able to use the machinery of amoeba to multiply, or whether amoebae were able to limit their proliferation. Seven strains belonging to the species *L. longbeachae*, *L. anisa*, *L. bozemanae*, *L. taurinensis,* and *L. dumoffii* were cocultured with three amoebae, *Acanthamoeba castellanii*, *Willaertia magna* T5(S)44, and *Willaertia magna* C2c Maky, at two temperatures, 22 and 37 °C. We found that at 22 °C, all amoebae were able to phagocytose the seven Lnp strains and to avoid intracellular development, except for *L. longbeachae*, which was able to multiply inside *W. magna* T5(S)44. At 37 °C, four Lnp strains were able to hijack the machinery of one or two amoebae and to use it to proliferate, but none were able to multiply inside *W. magna* C2c Maky.

## 1. Introduction

The genus *Legionella* currently comprises more than 60 species (https://lpsn.dsmz.de/genus/legionella) (accessed on 18 October 2021) [1], of which more than half have been shown to be pathogenic to humans [2,3,4]. These bacteria occur naturally in freshwater (lakes and rivers) and wet soils. From the natural environment, the bacteria colonise anthropogenic infrastructures, especially those containing stagnant water at a temperature between 25 and 45 °C. Free-living *Legionella* have difficulty surviving in the environment because of their nutritional requirements. They develop in biofilms or within protists such as ciliates and amoebae [5,6]. The presence of biofilm and certain materials such as iron, zinc, and aluminium favour their survival [7].

Free-living amoebae (FLA) are ubiquitous protozoa, which have been detected in a variety of environmental and manmade habitats, including freshwater, seawater, soil, air-conditioning units and drinking water treatment plants [8,9,10,11,12,13,14,15]. In their natural environment, FLA are an essential component of microbial communities, regulating bacterial populations by feeding on them, but they can also sometimes serve as hosts, vehicles, and reservoirs for pathogenic amoeba-resistant bacteria (ARB), including *Legionella* species [16,17,18]. 

*Legionella* have developed mechanisms to invade FLA by inducing "coiled phagocytosis" to escape digestion by inhibiting the fusion of the phagosome with the lysosome [6,19]. After intracellular replication, *Legionella* progressively invade the cytoplasm, leading to lysis of the amoeba and the release of mobile *Legionella*. In some cases, the amoeba also releases vesicles of 1–5 µm in diameter containing the *Legionella* [16,20,21], allowing their survival for several months [22]. When aerosols filled with bacteria are inhaled, *Legionella* can infect alveolar macrophages and epithelial cells [23]. It is suspected that, when an amoebic membrane surrounds them, it promotes their fusion with the membrane of pulmonary epithelial cells, increasing the risk of infection [20]. It has been shown that growth within FLA, especially in *Acanthamoeba*, protects *Legionella* from adverse environmental conditions, because their cysts are extremely resistant to environmental stresses such as desiccation and changes in pH, osmolarity, or temperature, and can even survive chlorination or other disinfection methods [24,25,26]. Amoebic passage also enhances, in some cases, the virulence of *L. pneumophila* [27,28], but it has been demonstrated that *Willaertia magna* C2c Maky did not [29]. Moreover, this amoeba proved to efficiently resist *L. pneumophila* [30,31,32].

In 2019 in Europe, 11,298 cases of legionellosis were detected [33]. A recent study estimated that the actual number of cases of Legionnaires’ disease may be 1.8 to 2.7 times higher than that reported [34]. Part of the explanation for this lies in the detection method used and the designation of confirmed cases. A urinary antigen test is often used to diagnose legionellosis, but it is not sensitive enough [35]. Although not time-consuming and easily implemented, this test only detects *Legionella pneumophila* serotype 1 (*Lp1*). Moreover, in the vast majority of cases, the diagnosis is based solely on antigenicity. The result of a positive PCR test will be designated as a probable case, but not as a confirmed case [36]. Many studies demonstrate that legionellosis cases were related to *Legionella* non-*pneumophila* (Lnp) strains and that these strains are frequently isolated in the environment [2,37,38,39,40,41,42,43]. For example, Steege and Moore detected by means of qPCR on the *mip* gene the presence of *Legionella* in 95% of samples, but *L. pneumophila* was only detected in 1.8% of samples [44]. In Japan from 2008 to 2016, *Lp1* was responsible for 81% of cases, but Lnp species such as *L. bozemanae*, *L. dumoffii,* and *L. longbeachae* were also isolated [37]. A recent study, using qPCR on the *mip* gene in hospital and community samples, demonstrated an increased prevalence of the Lnp species (84.1%) compared to *L. pneumophila* (15.9%) in the region of Bologna (Italy) [45]. 

Considering these data, the aim of this study was to evaluate the intracellular behaviour of seven Lnp strains belonging to the species *L. longbeachae*, *L. anisa*, *L. bozemanae*, *L. taurinensis,* and *L. dumoffii* in coculture with three FLA, *Acanthamoeba castellanii*, *Willaertia magna* T5(S)44, and *Willaertia magna* C2c Maky, to discover whether Lnp strains have the same intra-amoebal multiplicative properties as *L. pneumophila* strains. To compare our results with a previous study on three *L. pneumophila* strains [32], two temperatures were considered, 22 and 37 °C.

## 2. Results

### 2.1. L. Non-pneumophila Survival in an SCYEM Medium

The survival of seven L. non-pneumophila (Lnp) strains in the coculture medium was evaluated at 22 and 37 °C. 

**Figure 1:**
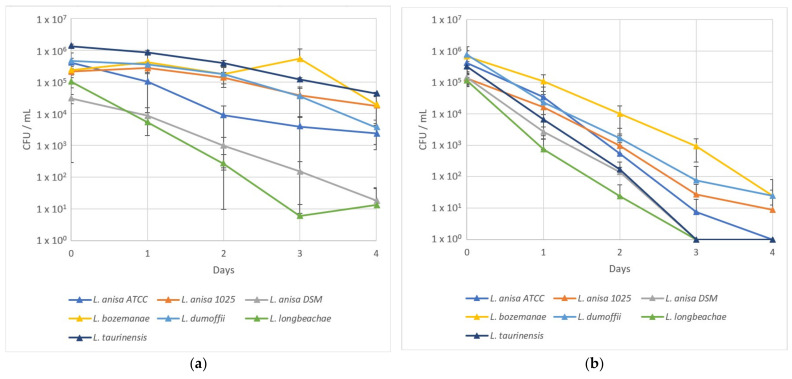
*L.* non-*pneumophila* survival in an SCYEM medium at 22 (**a**) and 37 °C (**b**). Results are expressed as the mean ± standard deviation (SD).

The seven bacterial strains had a better survival capacity at 22 °C than at 37 °C in the SCYEM medium (Figure 1a,b, respectively). 

At 22 °C, *L. longbeachae*, *L. anisa* ATCC, and *L. anisa* DSM decreased 100-fold after two days, whereas *L. bozemanae*, *L. taurinensis*, *L. dumoffii,* and *L. anisa* 1025 were only reduced 2-fold. After four days, *L. longbeachae* and *L. anisa* DSM decreased to 13 and 18 CFU/mL, respectively, whereas the five other strains were able to maintain levels between 2.4 × 10^3^ and 4.2 × 10^4^ CFU/mL (Figure 1a).

At 37 °C, *L. longbeachae*, *L. anisa* DSM, and *L. taurinensis* were not detectable on the plate after three days, nor *L. anisa* ATCC at day 4. The three other strains, *L. bozemanae*, *L. dumoffii,* and *L. anisa* 1025, decreased to 25 ± 12, 24 ± 57, and 9 ± 13 CFU/mL, respectively (Figure 1b). 

### 2.2. Amoeba Survival

The SCYEM medium allows the survival of the three amoebae during the four days of experimentation at 22 and 37 °C (Figure S1). Survival of the three amoebae in the presence of bacteria was evaluated for four days at 22 and 37 °C in an SCYEM medium.

**Figure 2:**
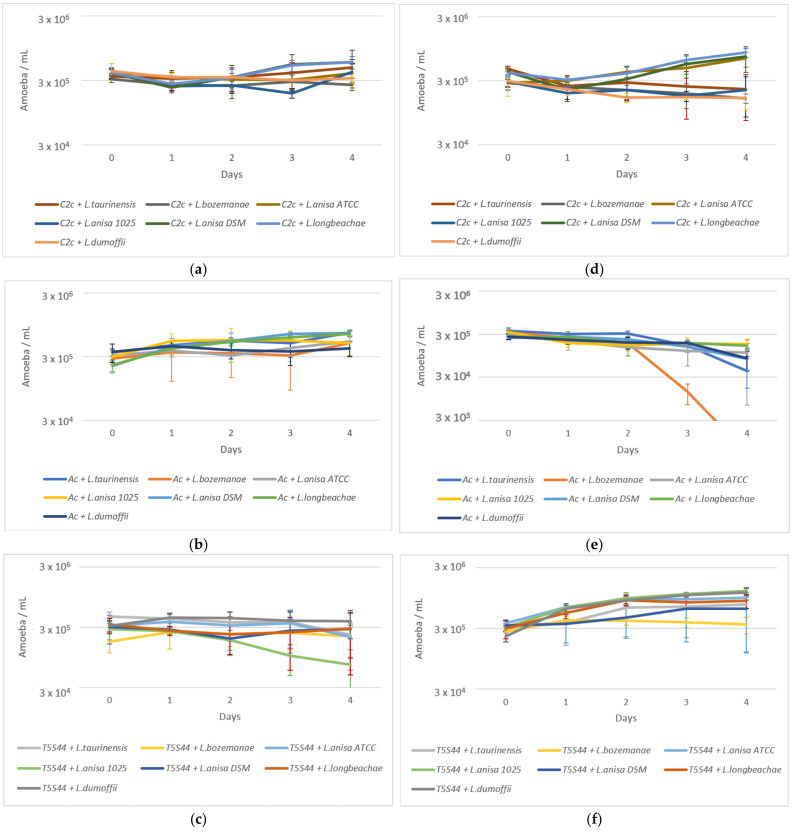
Amoeba survival at 22 (**a**–**c**) and 37 °C (**d**–**f**) in an SCYEM medium with the seven *L.* non-*pneumophila* strains. C2c: *W. magna* C2c Maky (**a**,**d**); Ac: *A. castellanii* (**b**,**e**); T5S44: *W. magna* T5(S)44 (**c**,**f**). Results are expressed as the mean ± SD.

At 22 °C, the number of *W. magna* C2c Maky cells remained stable with intracellular Lnp strains until the end of the experiment on day four (Figure 2a), whereas at 37 °C, the number of cells increased in *L. anisa* ATCC, *L. anisa* DSM, and *L. longbeachae* and decreased by a factor of two in the four other strains (Figure 2d).

At 22 °C, *A. castellanii* was able to grow with the seven intracellular bacterial strains until the end of the experiment at day four (Figure 2b), whereas at 37 °C, the number of cells remained stable or decreased by less than ten-fold for all strains except *L. bozemanae*, where the number of cells decreased approximately 100-fold from 3.31 × 10^5^ to 5.01 × 10^2^ cells/mL (Figure 2e).

At 22 °C, the number of *W. magna* T5(S)44 cells remained stable with intracellular bacteria, except in *L. anisa* 1025, where the number of cells decreased from 2.80 × 10^5^ to 7.25 × 10^4^ cells/mL (Figure 2c). At 37 °C, the number of cells increased, except in *L. bozemanae*, where the number of cells remained stable (Figure 2f).

### 2.3. Coculture Experiments

Considering the death of the Lnp strains in SCYEM medium at 37 °C, the phagocytosis effect by the amoebae could not be easily evaluated. However, the potential of the bacterial strains to hijack the amoeba machinery could be determined because if bacterial multiplication occurs, it can only be attributed to its ability to use amoebae as bioreactors, as no bacterial multiplication in the culture medium occurred. 

In order to compare the behaviour of the three amoebae in the presence of the seven Lnp strains, an efficacy percentage was calculated according to the following formula (Equation (1)):

(1) $$E_{\mathrm{assay}}=\frac{\mathrm{CFU}\;\left(t0\right)-\mathrm{CFU}\;\left(t\right)}{\mathrm{CFU}\;\left(t0\right)}\;\times 100$$

where $\mathrm{CFU}\;\left(t0\right)$ is the number of intracellular bacteria at day zero and $\mathrm{CFU}\;\left(t\right)$ is the number of intracellular bacteria at day one, two, three, or four. 

To consider the bacterial decrease due to the culture medium effect, the same calculation was applied to the control conditions (bacteria in SCYEM without amoeba) (Equation (2)):

(2) $$E_{\mathrm{control}}=\frac{{\mathrm{CFU}}_{c}\;\left(t0\right)-{\mathrm{CFU}}_{c}\;\left(t\right)}{{\mathrm{CFU}}_{c}\;\left(t0\right)}\;\times 100$$

where ${\mathrm{CFU}}_{c}\;\left(t0\right)$ is the number of bacteria at day zero in the control flask and ${\mathrm{CFU}}_{c}\;\left(t\right)$ is the number of bacteria in control flasks at day one, two, three, or four.

The net efficacy percentage was calculated by subtracting the medium effect from the efficacy values of cocultures according to the following formula (Equation (3)):

(3) $$E_{f}=E_{\mathrm{assay}}-E_{\mathrm{control}}$$

where $E_{\mathrm{assay}}$ is the efficacy percentage of both the amoeba and the culture medium to decrease the number of intracellular bacteria; $E_{\mathrm{control}}$ is the percentage of bacteria killed by the culture medium in control conditions. $E_{f}$ is the net efficacy of amoeba on Lnp strains.

If $E_{f}$ is positive, it means that the amoeba was efficient, and consequently, bacterial death was increased by the presence of the amoeba, whereas a negative $E_{f}$ means that the number of living *Legionella* cells increased, indicating that it was able to multiply within the amoeba (as Lnp strains are not able to multiply in the SCYEM medium).

**Figure 3:**
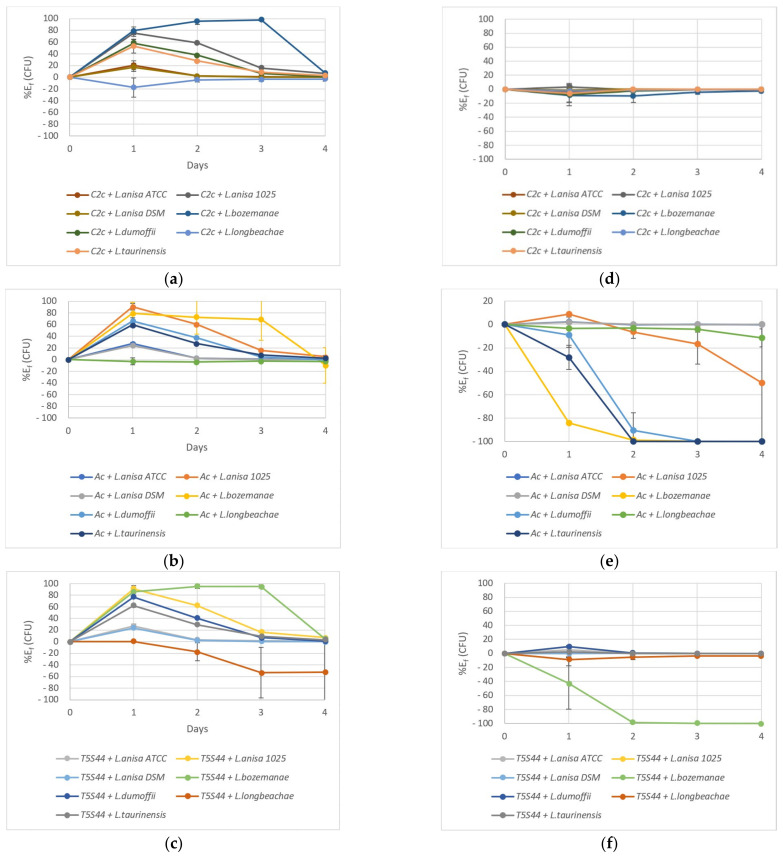
Efficacy of amoeba phagocytosis at 22 (**a**–**c**) and 37 °C (**d**–**f**) in an SCYEM medium on seven *L.* non-*pneumophila* strains. C2c: *W. magna* C2c Maky (**a**,**d**); Ac: *A. castellanii* (**b**,**e**); T5S44: *W. magna* T5(S)44 (**c**,**f**). Results are expressed as the mean ± SD.

At 22 °C, *W. magna* C2c Maky was efficient in phagocytosing and killing *L. taurinensis*, *L. dumoffii*, *L. anisa* 1025, and, in particular, *L. bozemanae*, with 98% efficacy after 72 h (Figure 3a). At 37 °C, the amoeba efficacy could not be proven, as the bacteria in the control condition died as fast as in the presence of *W. magna* C2c Maky (Figure 3d). However, at both temperatures, none of the seven *Legionella* were able to multiply in the presence of *W. magna* C2c Maky (Figure S2).

*A. castellanii* was efficient in phagocytosing and killing *L. taurinensis*, *L. dumoffii*, *L. anisa* 1025, and, in particular, *L. bozemanae*, with 69% efficacy after 72 h (Figure 3b). On the contrary, at 37 °C, no efficacy could be proven (Figure 3e). Moreover, *L. anisa* 1025 (Figure S2c), *L. bozemanae* (Figure S2d), *L. dumoffii*, (Figure S2e), and *L. taurinensis* (Figure S2f) were able to multiply inside *A. castellanii*. *A. castellanii* was completely invaded by bacteria from day two (Figure 3e). 

At 22 °C, *W. magna* T5(S)44 was efficient in phagocyting *L. taurinensis*, *L. dumoffii*, *L. anisa* 1025, and, in particular, *L. bozemanae*, with 95% efficacy after 48 h (Figure 3c), but it could not reduce the level of *L. longbeachae*, which was able to multiply inside the amoeba (Figure S2a). At 37 °C, no efficacy could be established as the bacteria in the control condition died as fast as in the presence of *W. magna* T5(S)44 (Figure 3f). Moreover, *L. bozemanae* was able to multiply within *W. magna* T5(S)44 cells that lost their efficacy from day two (Figure 3f) and allowed the bacterium to multiply (Figure S2d).

### 2.4. Microscopic Observations of Intracellular L. Non-pneumophila at 37 °C

Microscopic observations were performed at T_0_, T_0_ + 48 h, and T_0_ + 96 h. 

**Figure 4:**
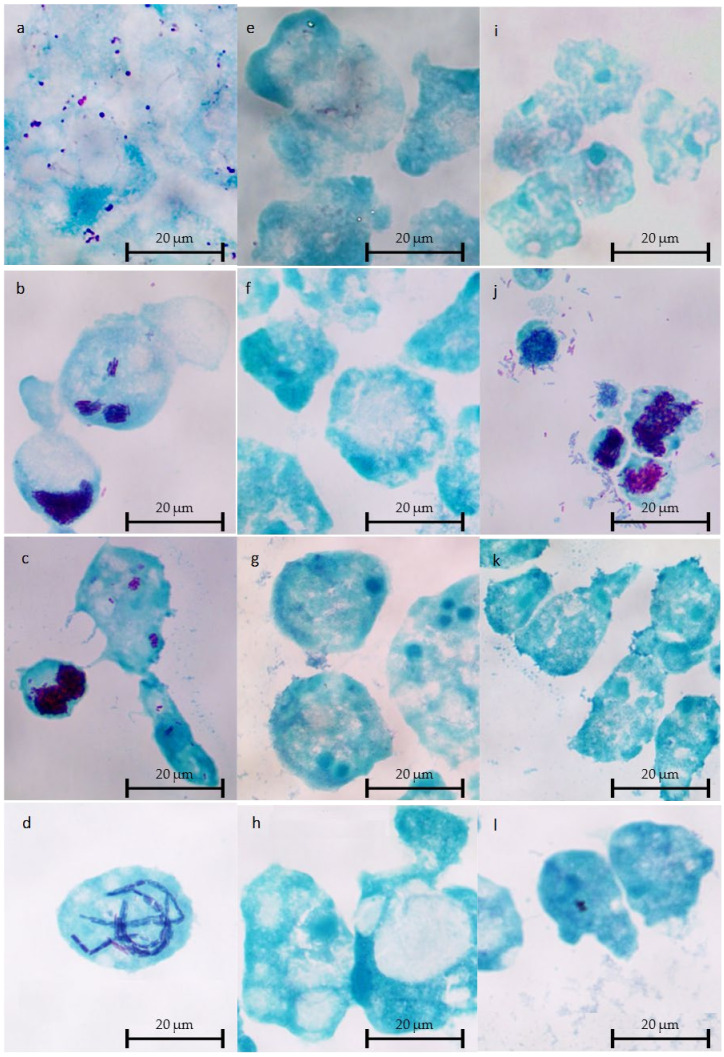
Optical microscopy observation using Gimenez staining of *A. castellanii* (**a**–**d**), *W. magna* C2c Maky (**e**–**h**), and *W. magna* T5(S)44 (**i**–**l**) infected with *L. anisa* 1025 (**a**,**e**,**i**), *L. bozemanae* (**b**,**f**,**j**), *L. dumoffii* (**c**,**g**,**k**), and *L. taurinensis* (**d**,**h**,**l**) after 96 h of coculture at 37 °C.

Intracellular multiplication at 37 °C detected by the plating approach were congruent with microscopic observations. Bacterial clusters were found in the cocultures of *A. castellanii* with *L. anisa* 1025 (Figure 4a), *L. bozemanae* (Figure 4b), *L. dumoffii* (Figure 4c), and *L. taurinensis* (Figure 4d), and of *W. magna* T5(S)44 with *L. bozemanae* (Figure 4j). No intracellular bacterial strain multiplication was observed within *W. magna* C2c Maky (Figure 4e–h).

## 3. Discussion

Even if *L. pneumophila* is responsible for most legionellosis outbreaks, *L.* non-*pneumophila* (Lnp) strains are also involved in legionellosis cases, and often their responsibility is under-recognized due to diagnostic bias [2,39,46]. Even though it is now well-known that among the approximately 60 species of *Legionella,* 50% are able to infect humans [47], the responsibility for 4% of legionellosis cases can still not be attributed to a known species [39]. An extensive detection of Lnp strains in water distribution systems demonstrated that 16% of the sampled water was contaminated with *Legionella*, and that Lnp strains were prevalent [48]. In the same way, an international survey demonstrated that 43 legionellosis cases among a total of 508 were due to Lnp strains, with the most prevalent being *L. longbeachae* [43]. Tools developed to study the behaviour of *L. pneumoniae* can be used to study Lnp. A clinical strain of *L. bozemanae* transformed with a GFP-expressing plasmid was able to infect and replicate within *A. castellanii* [49]. To increase knowledge of the replication of Lnp strains inside amoeba, we studied seven non *pneumophila* strains, three *anisa* isolates, and one isolate of strains *bozemanae*, *dumoffii*, *longbeachae,* and *taurinensis* (Table 1).

*L. anisa* ATCC 35291, isolated from a sink faucet in Illinois, USA, and strain DSM 17627 (ATCC 35292) from tap water in California, USA, were not associated with disease [50]. These two strains were not able to multiply within the three amoebae tested in this study. In contrast, *L. anisa* 1025 was isolated from a human lung and was responsible for the death of the patient [51]. This strain was able to hijack *A. castellanii* machinery at 37 °C and to multiply within the amoeba. Its closest relative is *L. pneumophila* strain Lens [52]. *L. anisa* is rarely encountered in legionellosis cases, being responsible for only 0.2% of cases [39,43]. However, it was responsible for an outbreak in California in 1988 [53] and was also isolated from a patient in Spain [42]. It is one of the most frequently isolated Lnp species from water systems [48]. 

*L. bozemanae* DSM 16523 (ATCC 33217) originated from a human lung tissue of a patient who died in 1959 (USA); its current name was proposed in 1980 [38,54]. *L. dumoffii* DSM 17625 (ATCC 33279) was isolated from a cooling tower in New York. For the last ten years, *L. bozemanae* and *L. dumoffii* were responsible for, respectively, 1 and 3% of legionellosis cases in New Zealand, and remained below 1% in other countries [39]. These two strains were able to multiply within *A. castellanii* at 37 °C, and *L. bozemanae* was also able to hijack the machinery of *W. magna* T5(S)44 at 37 °C.

*L. longbeachae* DSM 10572, originally designated as Long Beach 4, was isolated from transtracheal aspirates from a fatal case of pneumonia in 1980 [55]. In the case of *L. longbeachae* confirmed infections, its source was exclusively associated with soils, potting mixes, and composts [56]. It is responsible for 5% of legionellosis in the US and is endemic in New Zealand and Australia [2,57]. It was unable to multiply in *A. castellanii* and Mono Mac 6 cells [58]. This strain was not able to develop inside amoebae at 37 °C, but it could multiply within *W. magna* T5(S)44 at 22 °C.

*L. taurinensis* DSM 21897 (ATCC 700508) was isolated from a water sample of an oxygen bubble humidifier in a hospital in Turin (Italy), where nosocomial *Legionella* infection occurred [59,60]. It was able to multiply within *A. castellanii* at 37 °C, but not in the two *W. magna* strains. 

These data show that some Lnp strains can use amoebae for their own benefit. There is no general rule for this; this property is strain-dependent. The optimal temperature for growing *A. castellanii* is 28 °C. At 37 °C, *A. castelanii* showed limited growth, which may determine the interaction with *Legionella* species. However, none of the seven Lnp strains were able to multiply within *W. magna* C2c Maky, which is considered to be a non-permissive amoeba. Other amoebae have this property such as *Micriamoeba tesseris* [61] or environmental FLA isolates [62]. 

These results on seven Lnp strains reinforced the previous results on three *L. pneumophila* strains [32] to demonstrate that *W. magna* C2c Maky must have a very efficient process to fight amoeba-resistant bacteria and is a good candidate to be used as a biological biocide to treat industrial waters instead of, or along with, chemical treatments.

## 4. Materials and Methods

### 4.1. Microbial Cultures

*W. magna* C2c Maky (ATCC PTA-7824), *W.* *magna* T5(S)44 (ATCC 50036), and *A. castellanii* (ATCC 30010) were grown in an SCYEM medium as described in Hasni et al. [32]. Amoeba working suspensions (AWS) containing 3 × 10^5^ cells/mL were prepared.

Seven *Legionella* strains (Table 1) were grown and prepared as described for *L. pneumophila* strains in Hasni et al. [32]. Bacteria working suspensions (BWS) containing 3 × 10^7^ bacteria/mL were prepared.

### 4.2. Bacteria and Amoeba Survival in SCYEM Medium (Controls)

Survival of the 3 amoeba strains and 7 bacterial strains was monitored in SCYEM medium at both 22 and 37 °C for four days as described by Hasni et al. [32], except for the plating method. Samples were plated in duplicate with the easySpiral Pro apparatus (Interscience, Saint-Nom-la-Bretèche, France). Plates were incubated at 36 ± 2 °C, and CFUs were counted after 7 days with a Scan 500 reader (Interscience, Saint-Nom-la-Bretèche, France). Each condition was performed in triplicate and independently repeated (*n* = 6).

### 4.3. Coculture Assays

Amoeba and bacterial working solutions were mixed in equal quantity as described in Hasni et al. [32]. All flasks were left to stand for 2 h at 22 ± 2 °C or at 37 ± 2 °C to allow for amoeba–bacteria contact and the internalization of *Legionella* into amoebae. After the 2-hour contact step, each flask was gently shaken 10 times to detach amoeba cells, and the suspension was transferred into a 15 mL Flacon^®^ tube and centrifuged twice at 500× *g* for 10 min. The supernatant was removed and the cells were resuspended in 10 mL of fresh SCYEM medium. This step allowed for the removal of non-internalized (i.e., extracellular) *Legionella*. The suspensions were transferred into a new 25 cm^3^ flask and incubated at 22 ± 2 °C or at 37 ± 2 °C for four days. This time point corresponded to the T0 time point of the assay. Each condition consisted of three independent flasks and was repeated independently (*n* = 6).

### 4.4. Bacteria and Amoeba Quantifications in Coculture Assays from T0 to T96h 

At T0, T24h, T48h, T72h, and T96h, the supernatant was removed from each flask to detect only intracellular bacteria and replaced by 10 mL of sterile SCYEM according to Hasni et al. [32]. One millilitre was then sampled from each flask. Amoeba numbers were determined using a haemocytometer cell counting chamber method with Trypan blue. Intracellular *Legionella* CFUs were obtained after lysing amoeba with Triton™ X-100 at 0.02% *v*/*v* (final concentration) for 2 min according to Hasni et al. [32]. The samples were then serially 10-fold diluted in SCYEM and plated in duplicate with the easySpiral Pro apparatus. Plates were incubated at 36 ± 2 °C, and CFUs were counted after 7 days with a Scan 500 reader. Each condition was performed in triplicate and independently repeated (*n* = 6). 

### 4.5. Microscopic Observations of Cocultures

Cocultures were stained by means of the Gimenez technique [63,64] at T0, T48h, and T96h. Cocultures (0.5 mL) were centrifuged at 1000× *g* for 5 min, 0.45 mL of the supernatant was removed, pellets were resuspended in the remaining 50 µL, and 25 µL was deposited onto glass slides. After 5 min at room temperature, the cells were thermally fixed on a flame and then stained using the Gimenez technique. Briefly, each of the glass slides was stained with fuchsin solution for 3 min and washed with water. Then, the glass slides were stained with malachite green for 10–15 s and washed, and this step was repeated twice. Finally, the glass slides were allowed to dry at room temperature. Bacteria were stained in purple, and amoebae in blue. 

The observations were performed using a LEICA DM 2500 LED microscope (Leica Microsystemes SAS, Nanterre, France) under a 100× oil immersion objective.

## Figures and Tables

**Table 1:** List of *Legionella* strain studied.

Bacterial Name	Origin
*L. anisa* ATCC	ATCC 35291
*L. anisa* 1025	Clinical isolate
*L. anisa* DSM	DSM 17627/ATCC 35292
*L. bozemanae*	DSM 16523/ATCC 33217
*L. dumoffii*	DSM 17625/ATCC 33279
*L.* *longbeachae*	DSM 10572/ATCC 33462
*L. taurinensis*	DSM 21897/ATCC 700508

## Data Availability

Available on demand.

## Supplementary Materials

The following are available online at https://www.mdpi.com/article/10.3390/pathogens10101350/s1, Figure S1: Amoeba survival in SCYEM medium at 22 and 37 °C, Figure S2: Intracellular bacterial fate of bacteria.
