# Supplementary material for: Intracellular Behaviour of Legionella Non-pneumophila Strains within Three Amoeba Strains, Including Willaertia magna C2c Maky
Source: Pathogens. 2021 Oct 19;10(10):1350. doi: 10.3390/pathogens10101350 (PMC8538512; doi:10.3390/pathogens10101350)
Supplement: Supplementary file 1 [file pathogens-10-01350-s001.zip › pathogens-1395958-supplementary.pdf]

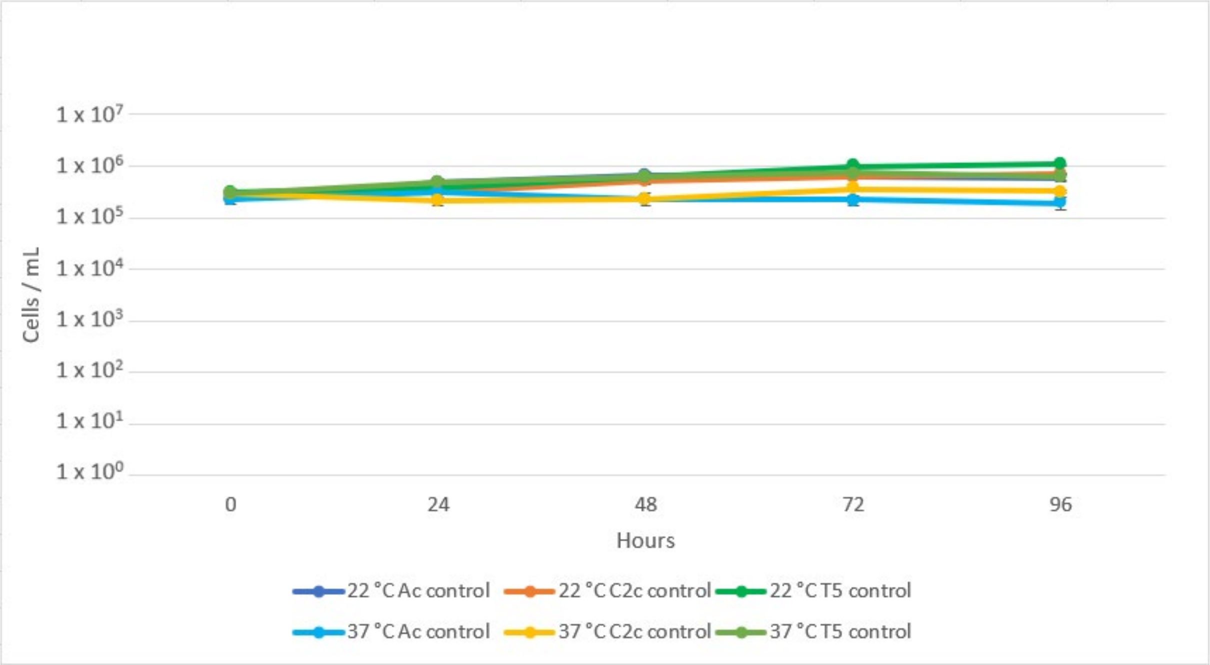

Figure S1. Amoeba survival in SCYEM medium at 22 °C and 37°C. Ac: *A. castellanii*; C2c: *W. magna* C2c Maky; T5S44: *W. magna* T5(S)44. Results are expressed as the mean ± SD.

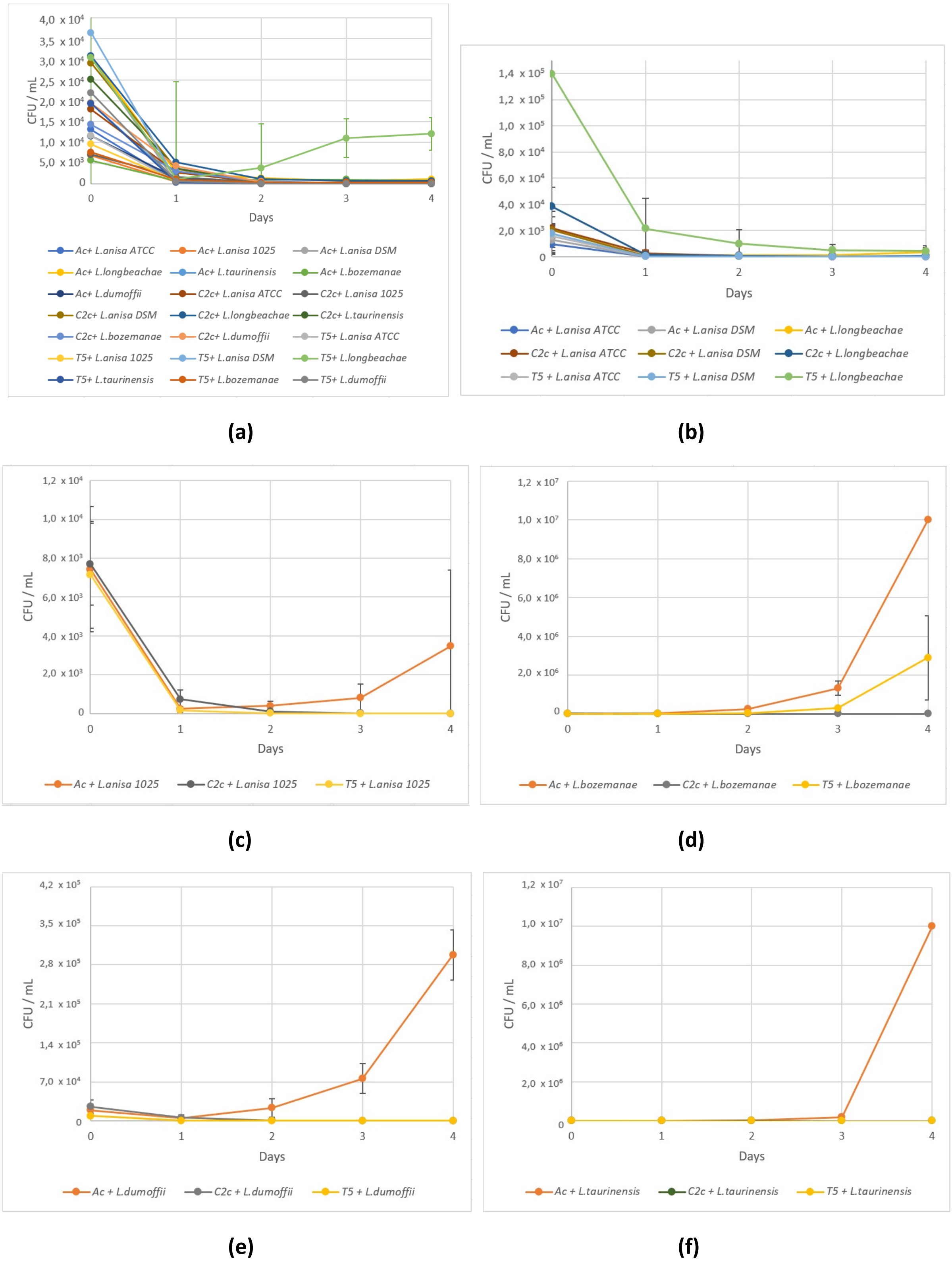

Figure S2. Intracellular bacterial fate of bacteria. **a:** all bacteria at 22°C; **b:** *L. anisa* ATCC, DSM and L. longbeachae at 37°C **c:** *L. anisa* 1025 at 37°C**, b:** *L. bozemanae* at 37°C**, d:** *L. dumoffii* at 37°C and **e:** *L. taurinensis* at 37°C. Ac: *A. castellanii* (**a-f**); C2c: *W. magna* C2c Maky (**a-f**); T5S44: *W. magna* T5(S)44 (**a-f**). Results are expressed as the mean ± SD.
